# Author Correction: Modulating co-translational protein folding by rational design and ribosome engineering

**DOI:** 10.1038/s41467-022-33270-4

**Published:** 2022-09-16

**Authors:** Minkoo Ahn, Tomasz Włodarski, Alkistis Mitropoulou, Sammy H. S. Chan, Haneesh Sidhu, Elena Plessa, Thomas A. Becker, Nediljko Budisa, Christopher A. Waudby, Roland Beckmann, Anaïs M. E. Cassaignau, Lisa D. Cabrita, John Christodoulou

**Affiliations:** 1 Institute of Structural and Molecular Biology, University College London, Gower Street, London, WC1E 6BT UK; 2 Gene Center and Department of Biochemistry, Ludwig-Maximilians-Universität München, Feodor-Lynen-Straße 25, 81377 Munich, Germany; 3 Institute of Chemistry, Technische Universität Berlin, D-10623 Berlin, Germany; 4 Faculty of Science, University of Manitoba, R3T 2N2 Winnipeg, MD Canada; 5 School of Crystallography, Birkbeck College, University of London, Malet Street, London, WC1E 7HX UK

**Keywords:** Solution-state NMR, Protein folding, Ribosomal proteins, Cryoelectron microscopy, Ribosome

Correction to: *Nature Communications* 10.1038/s41467-022-31906-z, published online 22 July 2022

The original version of this Article contained an error in the Acknowledgements section, which incorrectly omitted the following:

‘We acknowledge Diamond for access of the Cryo-EM facilities at the UK national electron bio-imaging centre (eBIC), proposals em20287 and bi26703, funded by the Wellcome Trust, Medical Research Council and BBSRC. We also wish to thank Dan Clare and all eBIC staff for data collection. Cryo-EM data for this investigation were also collected at ISMB EM facility (Birkbeck College, University of London) with financial support from the Wellcome Trust (202679/Z/16/Z and 206166/Z/17/Z). We thank Natasha Lukoyanova and Shu Chen for data collection and David Houldershaw for help with computing in data processing’.

This has been corrected in both the PDF and HTML versions of the Article.

