# Supplementary material for: Modulating co-translational protein folding by rational design and ribosome engineering
Source: Nat Commun. 2022 Jul 22;13:4243. doi: 10.1038/s41467-022-31906-z (PMC9307626; doi:10.1038/s41467-022-31906-z)
Supplement: Supplementary file 1 — Supplementary Information [file 41467_2022_31906_MOESM1_ESM.pdf]

# Modulating co-translational protein folding by rational design and ribosome engineering

## Supplementary information

Minkoo Ahn[1,6], Tomasz Włodarski[1,6], Alkistis Mitropoulou[1,6], Sammy H.S. Chan[1], Haneesh Sidhu[1], Elena Plessa[1], Thomas A. Becker[2], Nediljko Budisa[3,4], Christopher A. Waudby[1], Roland Beckmann[2], Anaïs M.E. Cassaignau[1], Lisa D. Cabrita[1,*] and John Christodoulou[1,5*]

[1]Institute of Structural and Molecular Biology, University College London, Gower Street, London WC1E 6BT, UK. [2]Gene Center and Department of Biochemistry, Ludwig-Maximilians-Universität München, Feodor-Lynen-Straße 25, 81377, Munich, Germany. [3]Institute of Chemistry, Technische Universität Berlin, D-10623 Berlin, Germany. [4]Faculty of Science, University of Manitoba, R3T 2N2 Winnipeg, MD, Canada. [5]School of Crystallography, Birkbeck College, University of London, Malet Street, London WC1E 7HX, UK.

*To whom correspondence should be addressed: Lisa D. Cabrita (LDC), l.cabrita@ucl.ac.uk, John Christodoulou (JC), j.christodoulou@ucl.ac.uk tel: +44 (0)20 76792375
[6]These authors contributed equally: Minkoo Ahn, Tomasz Włodarski, Alkistis Mitropoulou

| | | $23^{\Delta L}24^{\Delta L}$ Empty | $23^{\Delta L}24^{\Delta L}$ RNC | $23^{+L}$ empty | $23^{+L}$ RNC |
|---|---|---|---|---|---|
| Microscope | | FEI Titan Krios | FEI Titan Krios | FEI Titan Krios | FEI Titan Krios |
| Camera | | Falcon II | Falcon II | K3 | K3 |
| Voltage (kV) | | 300 | 300 | 300 | 300 |
| Pixel size (Å) | | 1.085 | 1.085 | 1.067/2 | 1.067/2 |
| Electron dose (e-/Å$^2$) | | 44 | 44 | 40.8 | 40.8 |
| Defocus range ($\mu$m) | | 0.5 - 2.5 | 0.5 - 2.5 | 0.5 - 2.5 | 0.5 - 2.5 |
| Particles after 2D (no.) | | 680,941 | 680,941 | 1,826,898 | 1,826,898 |
| Final particles (no.) | | 131,042 | 109,037 | 159,764 | 546,686 |
| Model composition | Protein residues | 5664 | 5668 | 3110 | 3143 |
| | RNA bases | 4551 | 4628 | 3006 | 3081 |
| Resolution (Å) | | 2.7 | 2.75 | 2.5 | 2.5 |
| FSC threshold | | 0.143 | 0.143 | 0.143 | 0.143 |
| Map CC (around atoms) | | 0.89 | 0.88 | 0.92 | 0.91 |
| Map CC (whole unit cell) | | 0.9 | 0.89 | 0.91 | 0.9 |
| Map sharpening B-factor (Å$^2$) | | -112.1 | -108 | -85 | -75 |
| RMS deviations | Bond lengths (Å) | 0.006 | 0.006 | 0.008 | 0.009 |
| | Bond angles (°) | 0.7 | 0.709 | 0.703 | 0.742 |
| Validation | MolProbity score | 2.2 | 2.1 | 2.02 | 2.06 |
| | Clash score | 15.7 | 13.1 | 11.41 | 10.8 |
| | Poor rotamers (%) | 0.09 | 0.06 | 0.08 | 1.25 |
| Ramachandran plot | Diallowed (%) | 0.25 | 0.2 | 0.07 | 0.1 |
| | Allowed (%) | 9.0 | 8.8 | 6.82 | 6.52 |
| | Favoured (%) | 90.75 | 91 | 93.12 | 93.38 |

**Supplementary Table 1.** Statistics for cryo-EM data collection, refinement and validation.

| Mutation | sgRNA | Donor DNA | Success rate (%) |
|---|---|---|---|
| $4^{\Delta L}$ | AAA AAA CCG TGG CGC CAG AA | CAG AAC GCC AGA TCG GGC TCT TGA TAG AAC CAG AAC GCG CCG GTT TTT TAC CGG AAC CAG TTA CTT CAG CAC GAG TCT T | 0 |
| $22^{\Delta L}$ | TGA TGT GGC TGG TGC GCT TC | TTT CGT AGA CGA AGG CCC GAG CAT GAA GCG CAT TAT GCG CAT ATT AAA GCG CAC CAG CCA CAT CAC TGT GGT TGT GTC CGA TCG CT | 2.8 |
| $23^{\Delta L}$ | AAA GGG AAA GTT AAA CGT CA | TTC TTT CAG GGT GAC GTA AGC TTT TTT CCA GTC GCT ACG ACG TTT AAC TTT CCC TTT AAC TAC CAG GGT GTT AAC GAC TTC GAC | 40.0 |
| $24^{\Delta L}$ | GGT TAA GAA ACA TCA GAA GC | TAC GTT GGA AAC CTG AAT AGC GGC TTC TTT TTC AAC GAT GCC ATG TTT CTT AAC CAG GTT GAT ACC TTC AAC AAT | 69.2 |
| $23^{+L}$ | AAA GGG AAA GTT AAA CGT CA | TTC TTT CAG GGT GAC GTA AGC TTT TTT CCA GTC GCT ACG ACG ACC GCT GGT GCG AAT CAC GCG GCC GCC GCT GCG CAC GGT CAT CTG GCG AAT TTT CCC TTT AAC TAC CAG GGT GTT AAC GAC TTC GAC TTC GAC TTC | 44.4 |
| $24^{+L}$ | GGT TAA GAA ACA TCA GAA GC | AAA CCT GAA TAG CGG CTT CTT TTT CAA CGA TGC CAC CCG GCA CGA TCT GCG TCC CAC CCA TGC GCG ACG AGT CCT TGA TCG GCT TTG GCT TCT GAT GTT TCT TAA CCA GGT TGA TAC CTT CAA CAA TG | 25.0 |

**Supplementary Table 2**. Single guide RNA (sgRNA) and single-stranded donor DNA (ssDNA) used for loop modification using homologous directed repair by CRISPR/Cas9. Success rate was calculated by the ratio of the number of positive colonies out of total number of colonies screened by colony PCR.

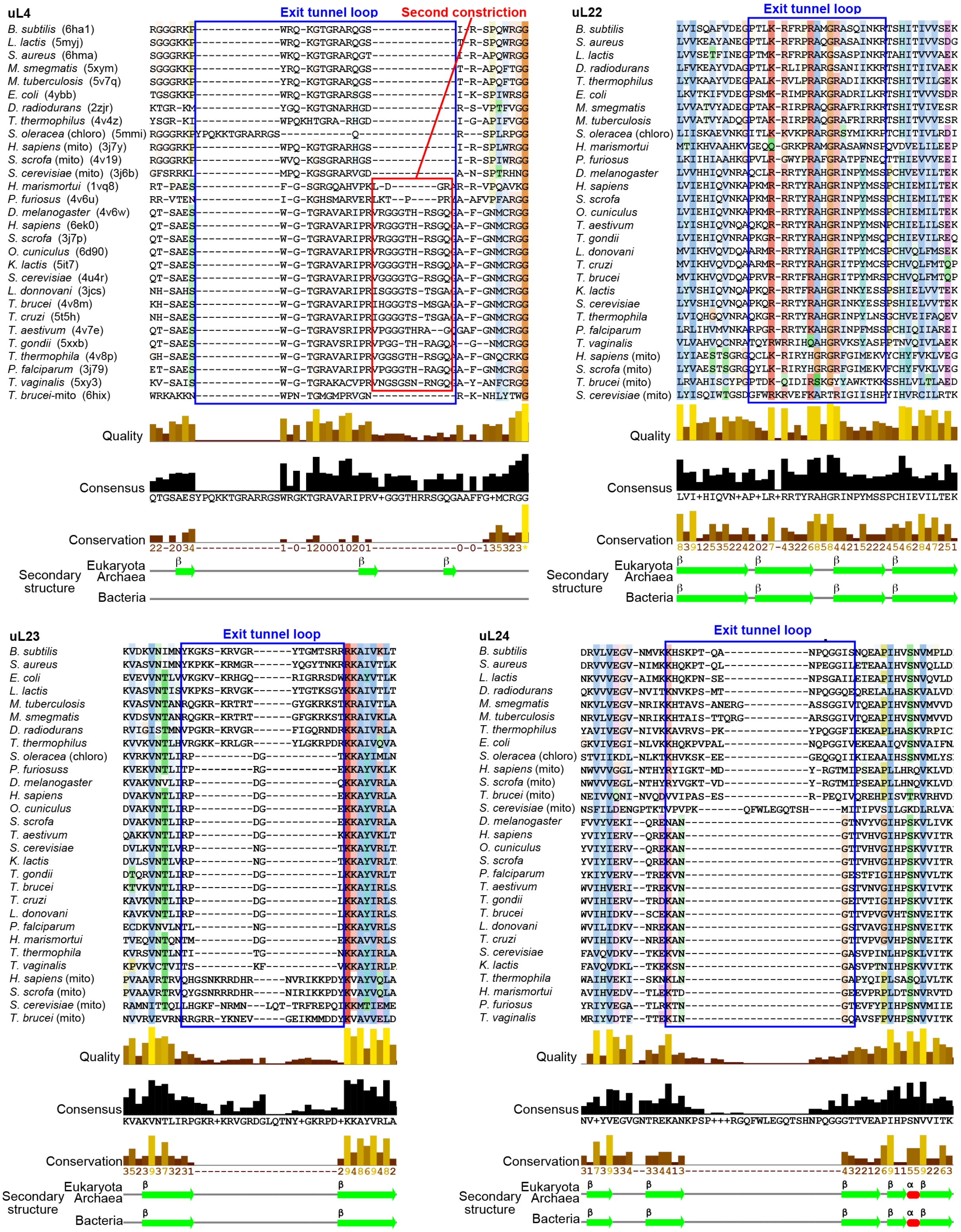


**Supplementary Figure 1. Structure-based multiple sequence alignments of the loops of the exit tunnel proteins (uL4, uL22, uL23 and uL24).** Shown are alignments from bacterial, eukaryotic and archaeal ribosomes and also include sequences from four mitochondrial and one chloroplastic ribosomes for comparison. The tunnel loop sequences are shown within the blue rectangle. Below each of the alignments are the extent of quality of alignment, sequence conservation and secondary structure prediction. The PDB id of the ribosome structure from each organism used for the alignment is shown next to the name of the organism in the uL4 sequences.

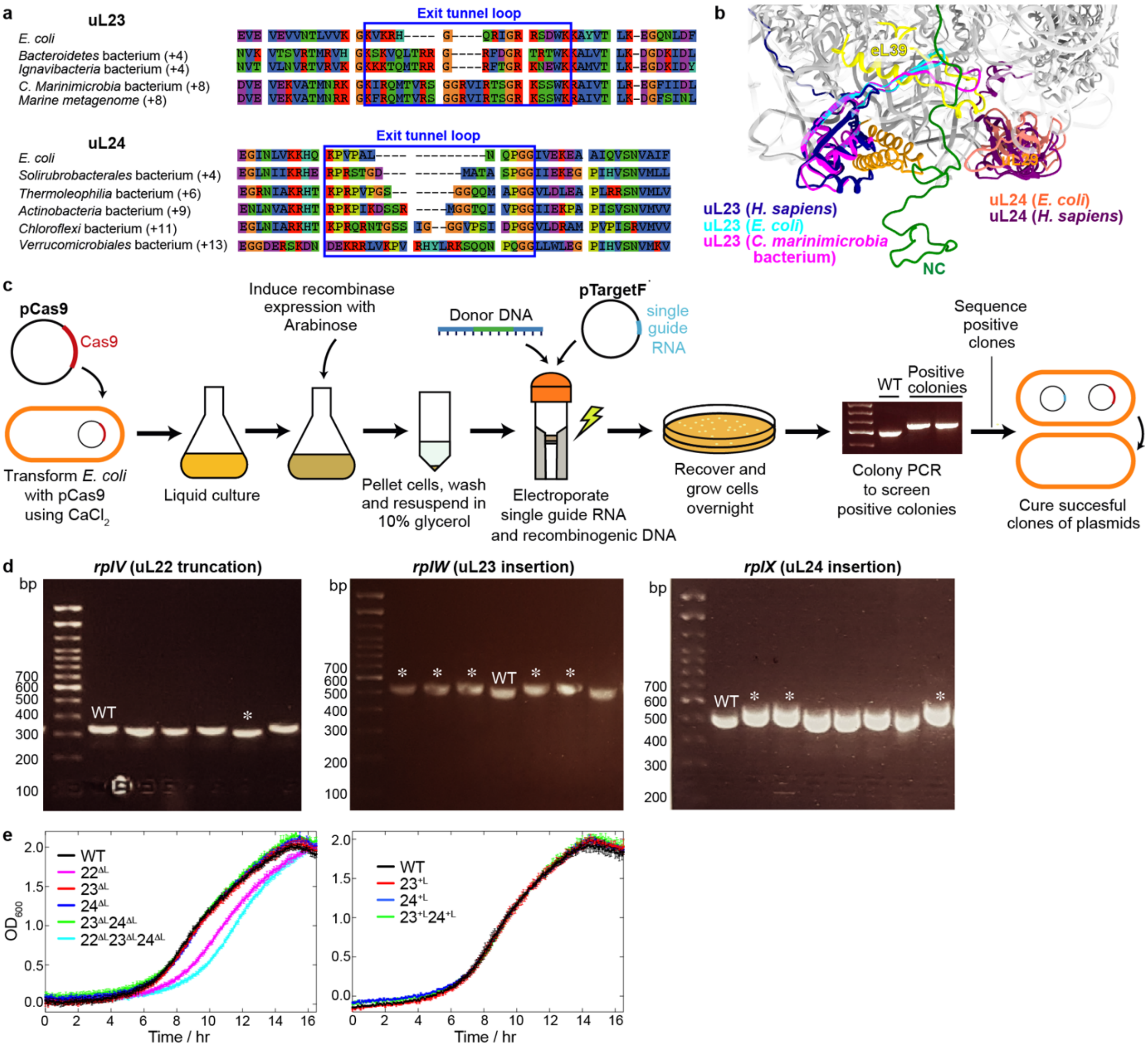


**Supplementary Figure 2. Design and engineering of *E. coli* ribosome with exit tunnel protein loop modifications by CRISPR-Cas9 genome editing. a**, Sequence alignments of uL23 and uL24 loop by MAFFT[1] for *E. coli* and bacterial sequences with long loops. **b**, Structure of 70S *E. coli* ribosome with bacterial and eukaryotic uL23 and uL24 and eukaryotic protein eL39. The space in the tunnel generated by the shorter uL23 loop of *H. sapiens* (dark blue) relative to that of *E. coli* (cyan) is filled by an additional eukaryotic protein eL39 (yellow). The longer uL23 loop sequence of *C. marinimicrobia* bacterium (magenta, modelled by Modeller[2]) and shorter uL24 loop sequence of *H. sapiens* (purple) were used for generating the chimeric mutant ($23^{+L}24^{\Delta L}$) of 70S ribosome to mimic eukaryotic 80S ribosome. **c**, A schematic of the CRISPR-Cas9 engineering workflow. Cells expressing Cas9 were cultured in LB and following induction of recombinase genes were prepared for electroporation by washing. Cells were then recovered, plated and grown for 24-48 hr and screened for positive colonies. Successful clones were sequenced and cured of plasmids. **d**, Representative agarose gels of colony PCRs of ribosomal protein genes, ∗ represents positive colonies and WT is the negative control. *rplV* (uL22, 24 bp deletion), *rplW* (uL23, 24 bp insertion), *rplX* (uL24, 27 bp insertion) show down-shifted or up-shifted positive colonies on the gel compared to the WT. More than 50 colonies are screened for each variant and the success rates are shown in (**c**). **e**, Monitoring the growth of WT and CRISPR-modified *E. coli* cells over time. Time courses measuring *E. coli* growth at $OD_{600nm}$ in M9 minimal media at 30 °C. Plotted are WT (black line) alongside the *E. coli* strains with modified ribosomes: (left) truncation mutants and (right) insertion mutants. All data are the average of three replicates with error bars showing the standard deviation.

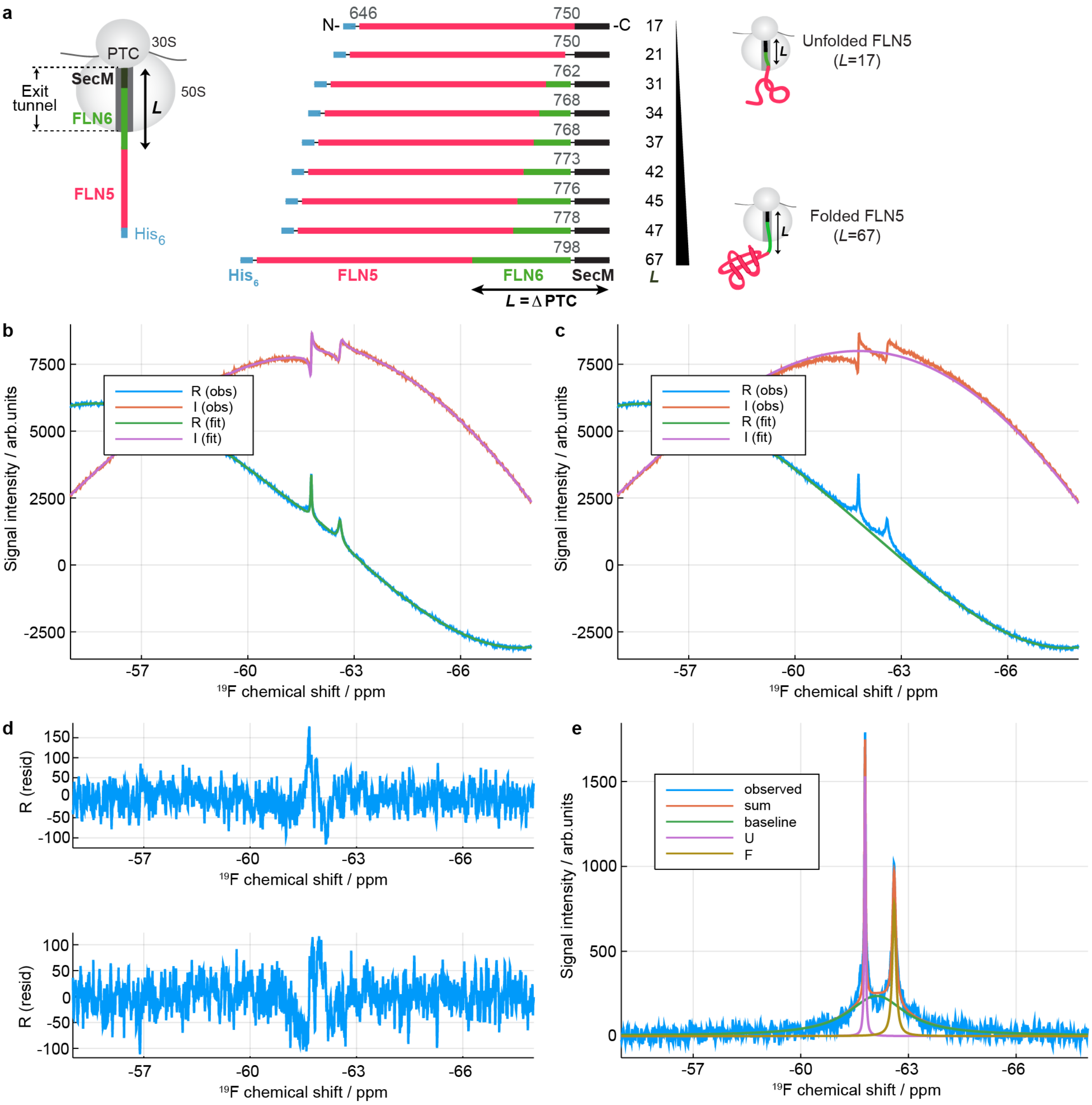


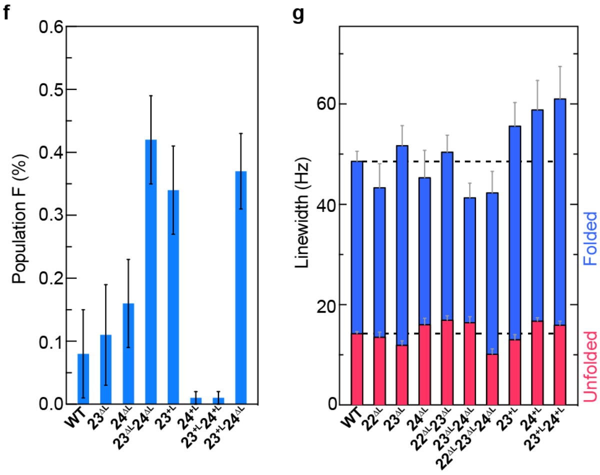


h

| | L=31 | | L=34 | |
|---|---|---|---|---|
| | $\Delta G_{F\text{-}U}$ | $\Delta\Delta G_{F\text{-}U}$ | $\Delta G_{F\text{-}U}$ | $\Delta\Delta G_{F\text{-}U}$ |
| WT | 1.50 ± 0.62 | | -0.21 ± 0.03 | |
| $23^{\Delta L}$ | 1.25 ± 0.44 | -0.25 ± 0.80 | -0.39 ± 0.07 | -0.18 ± 0.09 |
| $24^{\Delta L}$ | 0.99 ± 0.26 | -0.51 ± 0.72 | -0.36 ± 0.08 | -0.14 ± 0.10 |
| $23^{\Delta L}24^{\Delta L}$ | 0.19 ± 0.11 | -1.31 ± 0.68 | -0.55 ± 0.07 | -0.34 ± 0.09 |
| $23^{+L}$ | 0.41 ± 0.18 | -1.09 ± 0.70 | -0.40 ± 0.06 | -0.18 ± 0.08 |
| $24^{+L}$ | 2.76 ± 6.00* | 1.26 ± 0.86* | 0.07 ± 0.11 | 0.28 ± 0.12 |
| $23^{+L}24^{+L}$ | 2.76 ± 6.00* | 1.26 ± 0.86* | 0.06 ± 0.12 | 0.27 ± 0.13 |
| $23^{+L}24^{\Delta L}$ | 0.33 ± 0.11 | -1.17 ± 0.68 | N/A | N/A |
| $22^{\Delta L}$ | N/A | N/A | -0.20 ± 0.06 | 0.01 ± 0.08 |
| $22^{\Delta L}23^{\Delta L}$ | N/A | N/A | -0.33 ± 0.04 | -0.12 ± 0.07 |
| $22^{\Delta L}23^{\Delta L}24^{\Delta L}$ | N/A | N/A | -0.75 ± 0.12 | -0.54 ± 0.13 |

**Supplementary Figure 3. $^{19}$F 1D NMR spectroscopy and the time-domain fitting of the spectra. a**, Schematic depiction of the FLN5 RNC constructs used in NMR experiments ($^{19}$F, $^{15}$N and $^{13}$C NMR). Linker length (*L*) is the sum of SecM, FLN6 and the cloning residues (EL for *L*=31-67 and TSEF for *L*=21) between them as described previously[3]. **b-c**, Real and imaginary time-domain data from FID were fitted to two peaks – peaks for U and F states FLN NC and a broad baseline. The sum of the

peaks from the fitting (**b**) and the polynomial baseline of the FID (**c**) are shown with the recorded spectra. The stability of $^{19}$F RNC samples was monitored by the signal intensity over time, which showed no noticeable change during the first 8hr of measurement. The spectra recorded during this time were only used for data analysis to ensure that the observed signal solely come from the nascent chains. **d**, The residual of both real and imaginary data from the fitting. **e**, The observed spectrum and the peaks from fitting after baseline subtraction in (**c**). 5Hz line-broadening was used for presenting the data. **f**, Population of F state of RNCs at *L*=31 calculated from $^{19}$F NMR analysis. Errors are standard deviations (s.d.) calculated by Hamiltonian Monte Carlo analysis during the fitting of real and imaginary components of the FID simultaneously. **g**, The linewidths of U (red) and F (blue) peaks from $^{19}$F spectra of the WT and mutant RNCs (*L*=34). The dotted lines represent the linewidths of the U and F states of the WT RNC. Errors are s.d. calculated in the same way as for (f). **h**, Free energy of folding ($\Delta G_{F-U}$) of WT and mutant RNCs at *L*=31 and 34, and their differences ($\Delta\Delta G_{F-U}$) calculated from the populations of U and F NCs. All values are kcal/mol. *: For the variants that showed no F state signal, maximum $P_F$ of 1% was used to estimate $\Delta\Delta G_{F-U}$.

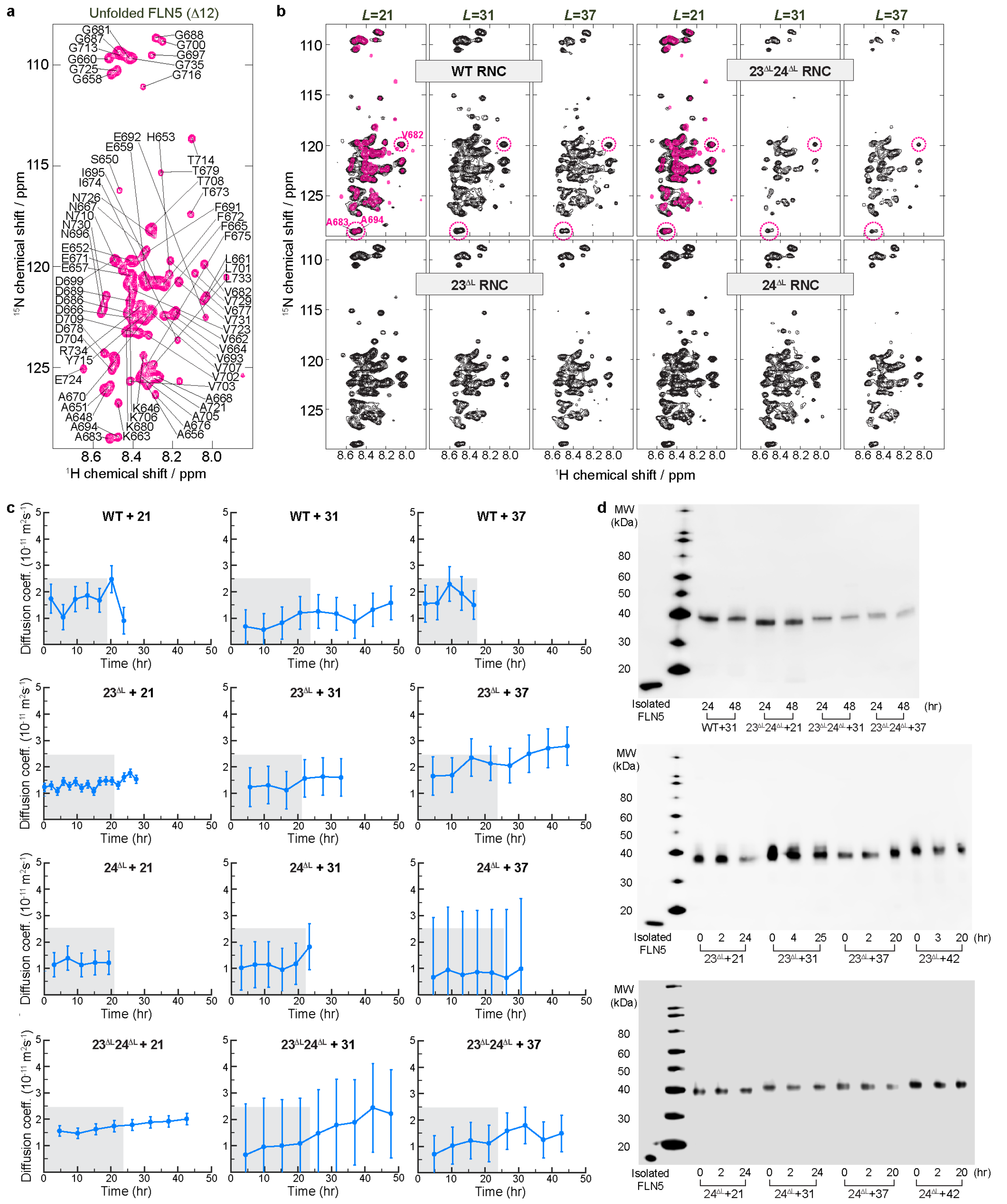


**Supplementary Figure 4. $^{1}$H-$^{15}$N correlation spectra of unfolded FLN5 on the WT and mutant ribosomes. a,** $^{1}$H-$^{15}$N correlation spectra of the isolated unfolded FLN5Δ12 (C-terminal truncation) with the assignments. **b**, Ribosome-bound unfolded FLN5 at various linker lengths (*L*=21-37) on WT, $23^{\Delta L}$, $24^{\Delta L}$ and $23^{\Delta L}24^{\Delta L}$ ribosomes. Isolated FLN5Δ12 spectrum is shown with the WT and $23^{\Delta L}24^{\Delta L}$ RNC spectra at *L*=21. Three unfolded peaks (V682, A683 and A694) that were used for intensity measurements are shown in magenta circles. All spectra were recorded at 10 °C and at a $^{1}$H frequency of 800MHz. The spectra were recorded at both 10 and 25 °C and showed no noticeable temperature effects on the population of the unfolded nascent chain. **c-d**, Monitoring the integrity of $^{15}$N-labeled RNCs by NMR spectroscopy (**c**) and western blot (**d**). **c**, SORDID NMR measurements of the unfolded FLN5. Diffusion coefficients were calculated using Stejskal-Tanner equation[3]. Grey rectangles represent the duration of time of which $^{1}$H-$^{15}$N SOFAST-HMQC data are included for analysis. No noticeable release was monitored from all RNCs up to 24 hr, and only the spectra recorded up to 24 hr were analysed. Errors are s.d. calculated by the spectral noise of each 1D spectrum. **d**, Anti-histidine western blot. All RNCs show no observable release of the NCs up to 48 hr on western blot, confirming

that the RNCs were intact during the time frame used for NMR analysis. Western blots are repeated at least twice.

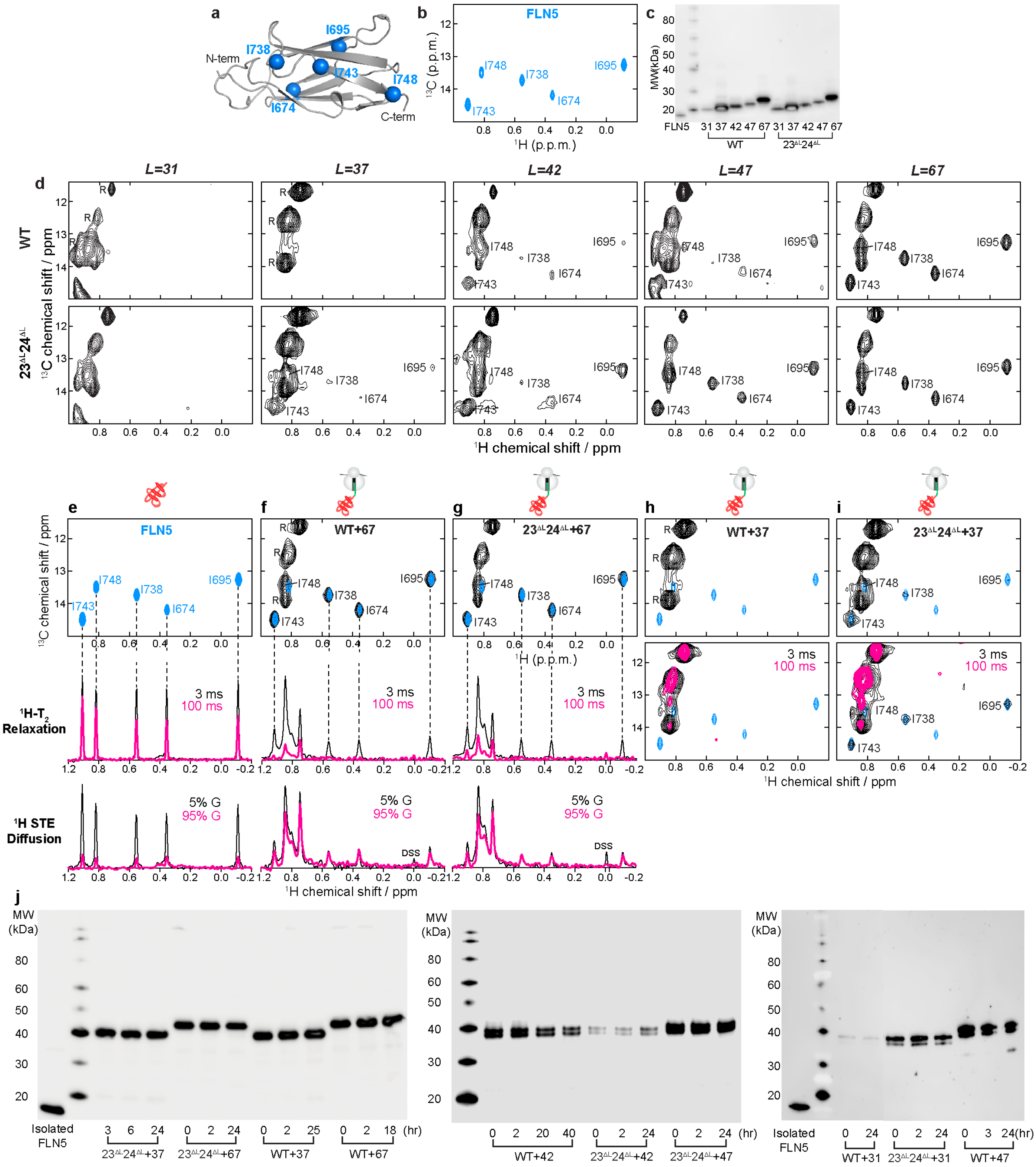

**Supplementary Figure 5. $^1$H-$^{13}$C ILE NMR spectra of folded FLN5 on the WT and 23$^{\Delta L}$24$^{\Delta L}$ ribosomes. a**, Crystal structure of folded FLN5 (PDB:1QFH). Blue spheres represent five isoleucine residues that are utilised as probes in $^{13}$C-labeled folded FLN5 spectra. **b**, $^1$H-$^{13}$C correlation NMR spectra of isolated folded FLN5. Five Ile peaks are shown with their assignment. **c**, Anti-histidine western blot of the released NCs from WT and 23$^{\Delta L}$24$^{\Delta L}$ RNC samples for $^1$H-$^{13}$C measurements in **d**. Samples were treated with RNase to release the NCs from the ribosome. The western blot of the NCs that are bound to the tRNA are in (**j**). **d**, $^1$H-$^{13}$C correlation NMR spectra of WT (upper row) and 23$^{\Delta L}$24$^{\Delta L}$ (lower row) RNCs. The folded Ile peaks above the noise level are shown with their assignment. The spectra are recorded at a $^1$H frequency of 800 MHz. **e-j**, Monitoring the integrity of the $^{13}$C-labelled WT and 23$^{\Delta L}$24$^{\Delta L}$ RNCs by NMR spectroscopy (**e-i**) and western blot (**j**). **e-g**, 1D $^1$H-$T_2$ relaxation and $^1$H STE Diffusion measurements of isolated FLN5 (**e**) and WT (**f**) and 23$^{\Delta L}$24$^{\Delta L}$ (**g**) RNCs at *L*=67. Isolated FLN5 shows slow transverse relaxation and fast translational diffusion due to its small molecular weight (~11kDa) (**e**). NCs bound to both ribosomes show the opposite characteristics – fast relaxation and

slow diffusion due to their attachment to the ribosome, the size of which is more than two orders of magnitude greater in its molecular weight (~2 MDa) than FLN5. **h**-**i**, 2D $^{1}H$-$T_2$ relaxation measurements of WT (**h**) and $23^{\Delta L}24^{\Delta L}$ (**i**) RNCs at *L*=37. WT RNC has no signal from the folded state as in 2D spectra in (**d**), hence no signal in 2D relaxation measurements (**h**). $23^{\Delta L}24^{\Delta L}$ RNC shows fast relaxation of NC (folded peaks of I743, I738 and I695 are broadened beyond detection at the second relaxation delay - 100ms) as in 1D relaxation measurements in (**f**) and (**g**) because of its attachment to the ribosome. (**j**) Anti-histidine western blot of the $^{13}C$-Ile NMR samples. All RNCs show no release of the NCs, confirming the intact RNCs during the NMR experiments. Western blots are repeated at least twice.

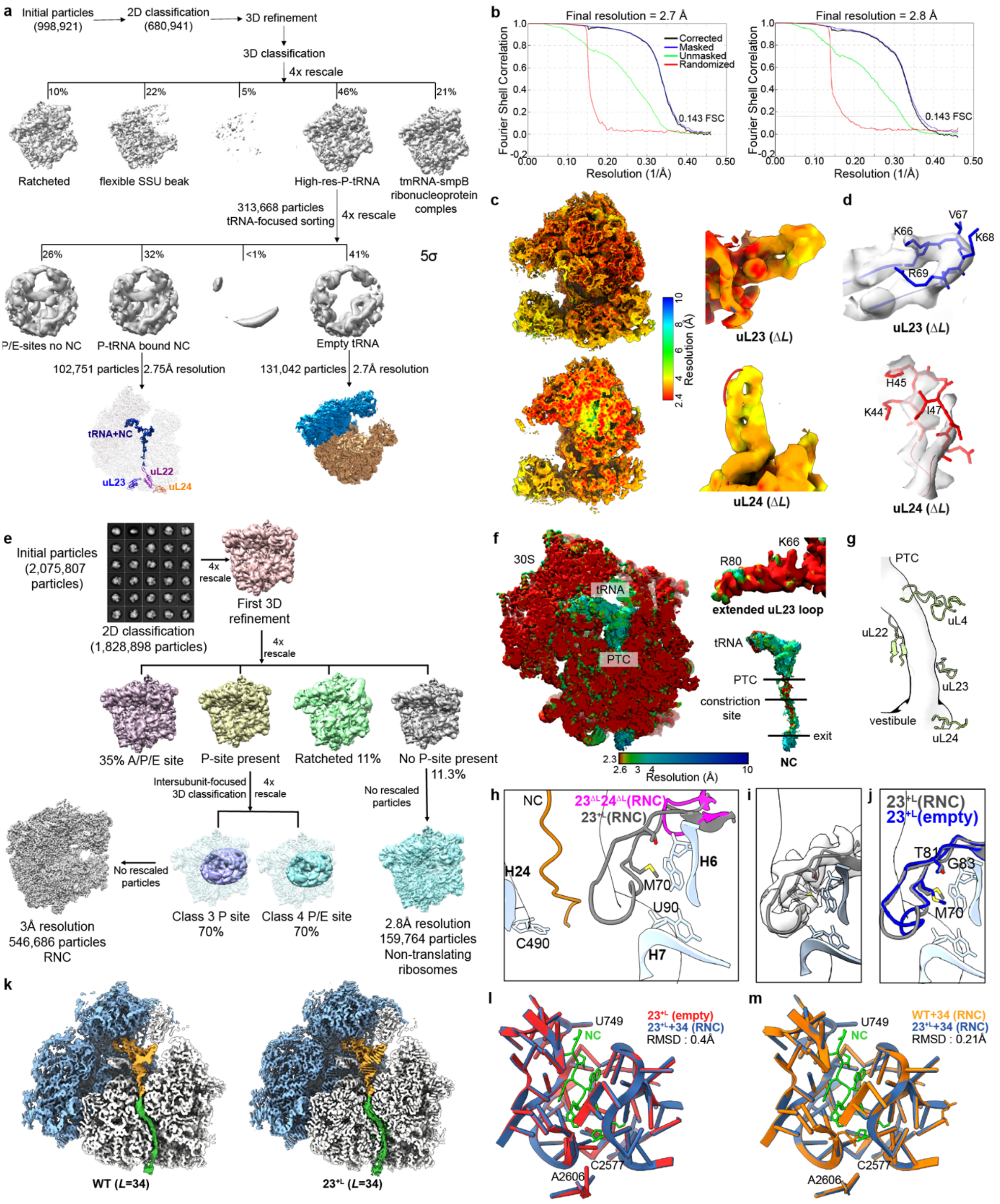


**Supplementary Figure 6. Classification, processing and local resolution of $23^{\Delta L}24^{\Delta L}$ and $23^{+L}$ ribosome by Cryo-EM. a-c**, $23^{\Delta L}24^{\Delta L}$ ribosome cryo-EM image processing and analyses. **a**, Image processing of the $23^{\Delta L}24^{\Delta L}$ ribosome. **b**, Fourier-shell correlation (FSC) curves of the refined final map of empty $23^{\Delta L}24^{\Delta L}$ ribosome (left) and P-tRNA RNC (right). **c**, Empty $23^{\Delta L}24^{\Delta L}$ ribosome EM density coloured by local resolution. **d**, The density of the uL23 and uL24 loops and the fitted amino acid residues. **e-j**, $23^{+L}$ ribosome reconstruction by cryo-EM**. e.** Image processing of the $23^{+L}$ datasets. **f**, $23^{+L}$ RNC EM density coloured by the local resolution. The local resolution of the density inside the tunnel ranges from 2.3Å (near the PTC) to 8Å (at the vestibule) and 8-10 Å (beyond the vestibule where the NC globular domain exits near H24 and uL24 loop). The overall ribosome structure is virtually identical to the empty $23^{+L}$ ribosome with the only local changes in the PTC nucleotides as expected. Right: EM density corresponding to the uL23 loop coloured according to the local resolution (top) and the isolated density of the tRNA-bound NC coloured according to the local resolution (bottom). **g**, The extended uL23 loop in the exit tunnel relative to other ribosomal protein loops. The extended loop points

away from the ribosomal tunnel. Ribosomal protein loops are shown in green and the tunnel wall is marked in black lines. **h**, Comparison of the extended uL23 loop (grey) with the truncated uL23 loop of the $23^{\Delta L}24^{\Delta L}$ RNC (*L*=37, magenta). No difference is observed in the environment near the loop. **i**, Comparison of the extended uL23 loop electron density with the modelled structure. **j**, Comparison of the extended uL23 loop on the empty $23^{+L}$ ribosome and $23^{+L}$ RNC. **k**, $23^{+L}$ and WT (*L*=34) RNC EM densities. P-tRNA and NC are coloured in orange and green, respectively. **l**-**m**, Comparison of the PTC structure between translating and non-translating $23^{+L}$ ribosomes (**l**) and WT and loop modified ($23^{+L}$) RNCs (**m**). No significant structural difference is observed at the PTC due to the NC stalling by SecM (**l**) and uL23 loop modification (**m**).

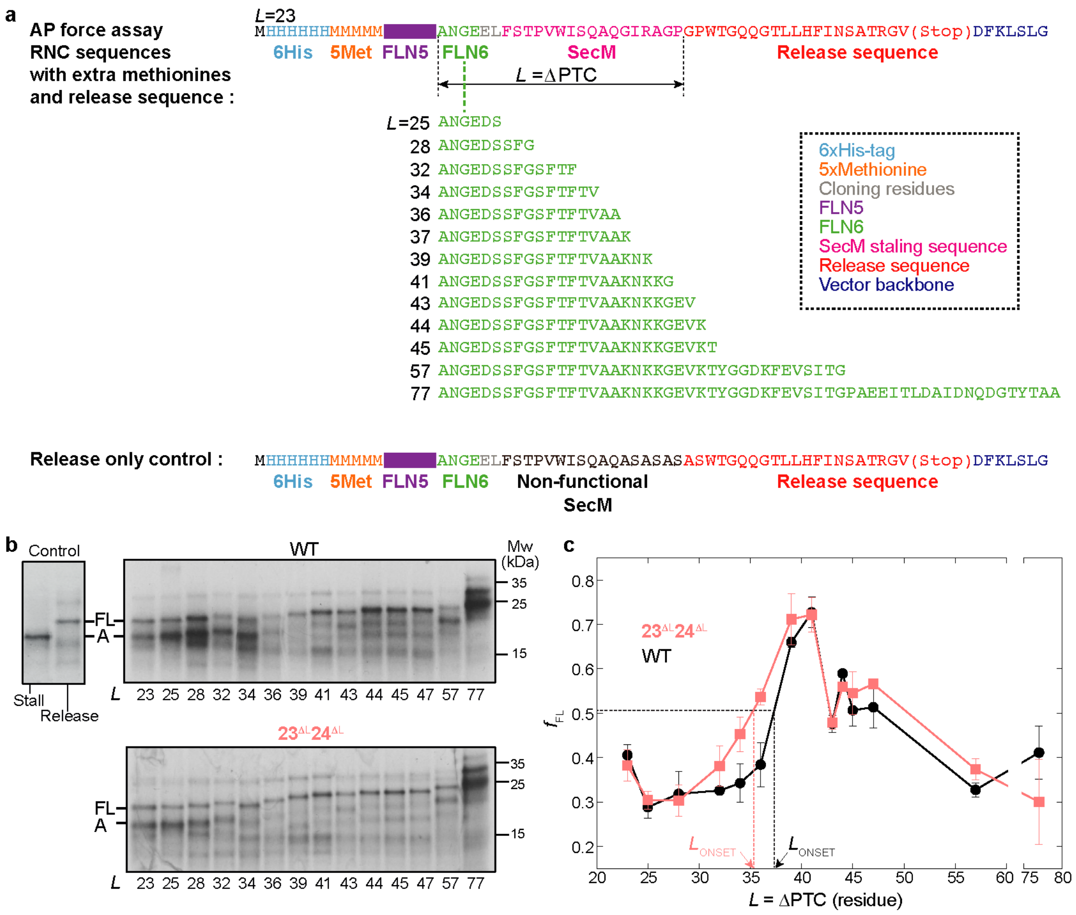


**Supplementary Figure 7. Arrest peptide (AP) force assay on the WT and $23^{\Delta L}24^{\Delta L}$ RNCs. a**, RNC sequences used for AP-force assay. The FLN5 RNC template was used to introduce 5 methionine after the His-tag and a release sequence following the SecM sequence. A release only control was created by replacing SecM with a non-functional SecM stalling sequence. **b**, Radiolabelled gel images of AP force assay reactions of FLN5 on WT (top) and $23^{\Delta L}24^{\Delta L}$ (bottom) ribosomes resolved on Bis-Tris gels with varying RNC linker length (*L*), indicated by numbers labelled for each lane. Stall only and release only control are shown on the left. FL and A indicate full length and arrested protein, respectively. **c**, Force profiles of FLN5 on WT (black circles) and $23^{\Delta L}24^{\Delta L}$ (red squares) ribosomes derived from densitometry analysis. The force profiles of FLN5 with increasing linker lengths show greater fraction of released full-length protein at *L*=26-41 on $23^{\Delta L}24^{\Delta L}$ ribosome, indicative of enhanced folding of FLN5. Folding onset ($L_{ONSET}$) was defined by the linker length (*L*) at which $f_{FL} = (f_{FL,max} + f_{FL,min})/2$. $L_{ONSET}$ of WT and $23^{\Delta L}24^{\Delta L}$ RNCs were approximately 35 and 37, respectively. Errors are s.d. deriving from ≥2 biological replicates.

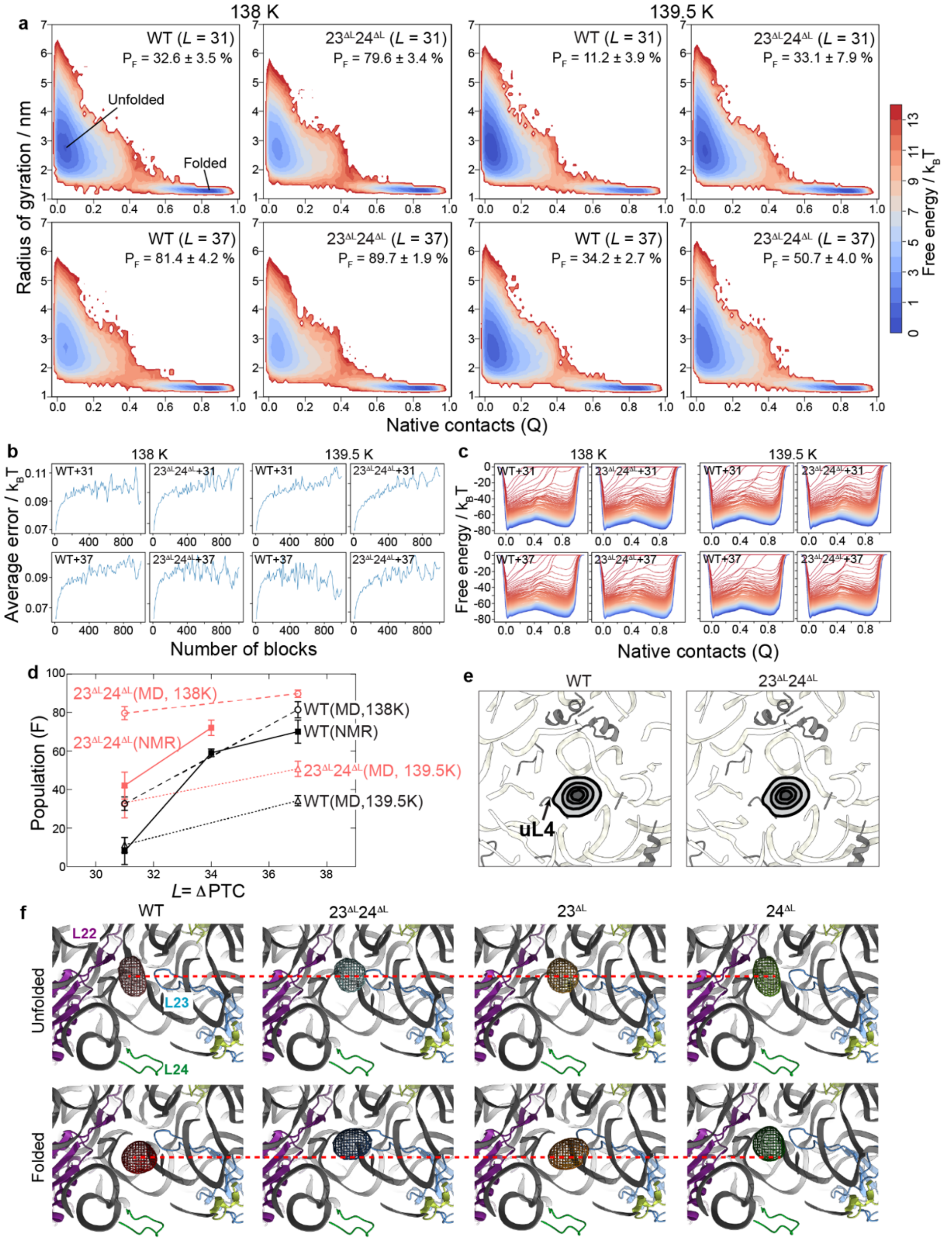


**Supplementary Figure 8. All-atom MD simulations of the WT and mutant RNCs.** **a**, 2D Free energy landscapes of the FLN5 nascent chain of WT and $23^{\Delta L}24^{\Delta L}$ RNCs at *L*=31, 37 at 138 and 139.5 K (simulation temperatures). The free energy was plotted as a function of radius of gyration and native contacts (Q). The folded population ($P_F$) for each RNC was calculated based on the free energy landscape by integrating the folded state minimum (Q>0.4). The simulations at two different temperatures show the same trend in the folded state populations (earlier folding of $23^{\Delta L}24^{\Delta L}$ RNC relative to the WT). **b**, Average error from block analysis for calculating the uncertainties of $P_F$ in **a**. **c**, Convergence of the free energy plotted against native contacts (Q). **d**, Comparison of $P_F$ from $^{19}$F NMR spectroscopy and all-atom MD simulations. Errors are s.d. deriving from fitting the NMR spectra (Fig. 2d and Supplementary Fig. 3f) or from the block averages of unfolded and folded states populations obtained from MD trajectory (**a**). **e**, Cross-sectional views of the probability density of the nascent chain ensemble with folded FLN5 (*L=37*) at the vicinity of the uL4 loop at the constriction site at 36.6 Å from the PTC. The densities are coloured in black corresponding to the SecM stalling sequence. **f**, Location

of the C-terminus of the FLN5 (residue 750) inside the tunnel at *L*=31. The most probable location of the last residue of the unfolded and folded FLN5 on the WT and truncation variant ribosomes from all-atom MD simulations are shown in mesh. The uL23 and uL24 loops of the WT ribosome are shown on the truncated ribosome variants for clarity. Red dashed line indicates the centre of the density of residue 750 of the WT RNC in the direction perpendicular to the exit tunnel.

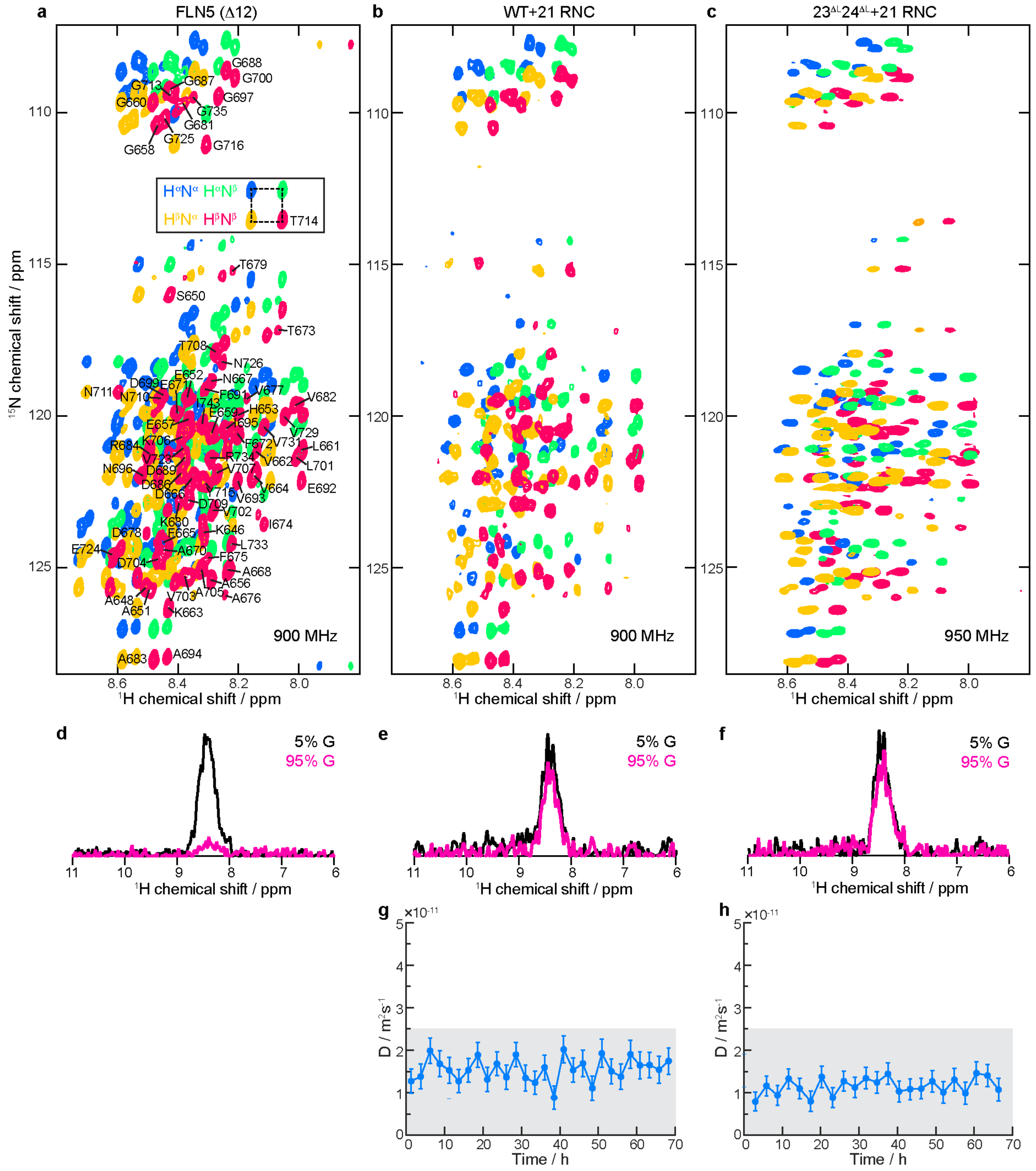


**Supplementary Figure 9. Cross-correlated relaxation (CCR) measurements of isolated unfolded FLN5(Δ12), WT and $23^{\Delta L}24^{\Delta L}$ RNCs (*L*=21) by BEST-TROSY-CCR NMR experiments**. **a-c**, Four spectra of TROSY and anti-TROSY peaks of each sample are over-laid and shown together for each sample. Blue, green, yellow and red peaks indicate the peaks of $H^{\alpha}N^{\alpha}$, $H^{\alpha}N^{\beta}$, $H^{\beta}N^{\alpha}$, $H^{\beta}N^{\beta}$ spin state, respectively[4]. **d-f**, $^{15}N$ SORDID diffusion measurements of FLN5Δ12, WT+21 and $23^{\Delta L}24^{\Delta L}$+21 RNCs at the beginning of the CCR experiments. Isolated unfolded FLN5 show fast translational diffusion – dramatically reduced signal intensity at the stronger gradient (95%G), whereas both WT+21 and $23^{\Delta L}24^{\Delta L}$+21 RNCs show slow diffusion of NC due to the NC tethered to each ribosome. **g-h**, Diffusion coefficients of WT+21 and $23^{\Delta L}24^{\Delta L}$+21 RNCs during the CCR measurements from $^{15}N$ SORDID experiments. Grey rectangles represent the duration of time in which the RNCs were intact and of which CCR data were used for the analysis of CCR rates. Both samples showed no release of FLN5 during the CCR measurements. Errors are s.d. calculated by the spectral noise of each 1D spectrum.

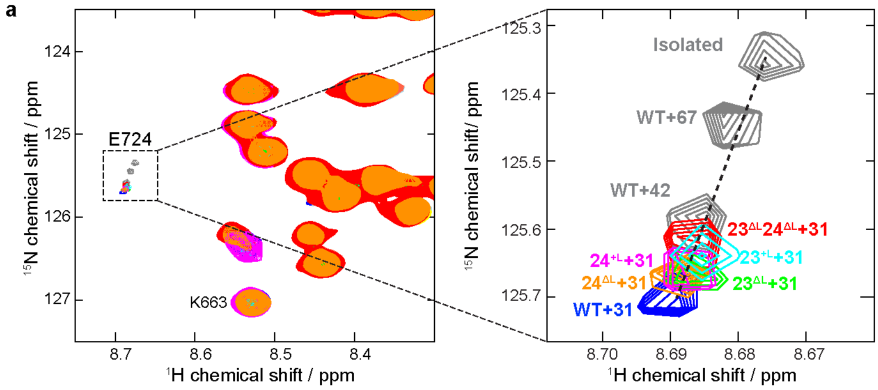


**b**

| RNC | P_Ub (%) | $\Delta G_{Binding}$ (kcal/mol) |
|---|---|---|
| WT+31 | 90.8 ± 0.6 | 1.28 ± 0.04 |
| $23^{\Delta L}$ | 81.4 ± 0.6 | 0.83 ± 0.02 |
| $24^{\Delta L}$ | 85.0 ± 0.6 | 0.97 ± 0.02 |
| $23^{\Delta L}24^{\Delta L}$ | 71.4 ± 0.6 | 0.51 ± 0.01 |
| $23^{+L}$ | 75.8 ± 0.6 | 0.64 ± 0.02 |
| $24^{+L}$ | 81.9 ± 0.6 | 0.84 ± 0.02 |
| WT+42 | 62.2 ± 0.6 | 0.28 ± 0.01 |

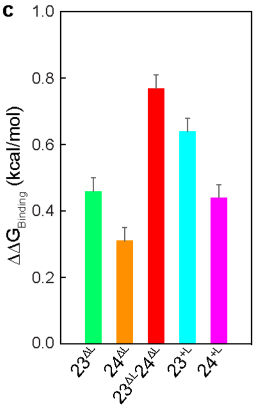


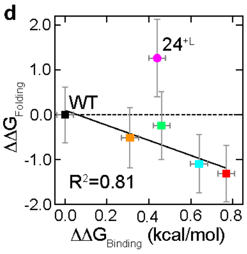


**Supplementary Figure 10. Binding of $A_3A_3$ FLN5 NC with the WT and mutant ribosomes assessed by $^{15}$N NMR a**, $^{1}$H-$^{15}$N cross-peaks of E724 of the $A_3A_3$ FLN5 on the WT (*L*=31, 42) and mutants RNCs (*L*=31). E724 resonance of the isolated unfolded protein is also shown, which was used as the chemical shift reference for the unbound unfolded nascent chain[4]. All spectra are recorded at a $^{1}$H frequency of 950 MHz. **b**, Population of the bound state of unfolded nascent chain (P_Ub) and free energy of binding ($\Delta G_{Ub\text{-}Uub}$) of each RNC estimated by E724 chemical shifts. **c**, Changes in the free energy of binding ($\Delta\Delta G_{Ub\text{-}Uub}$) of the mutant RNCs. **d**, Comparison of the folding and binding free energies of WT and mutant RNCs at *L*=31. Data points for the mutant RNCs were presented in the same colour as in **a** and **c**. $R^2$ was calculated from the linear regression analysis of the WT and the mutant RNC data points that showed the same tendency (negative correlation between the binding and folding free energies). Errors are s.d. calculated by propagating the s.d. of P_Ub and P_Uub.

## Original blots and gel images

Figure S2e

*rplV*

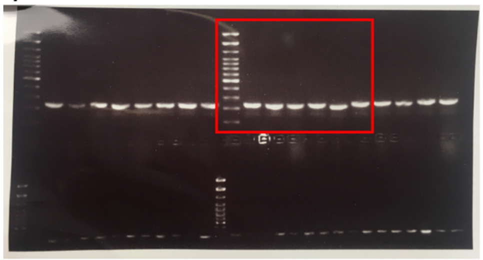

*rplX*

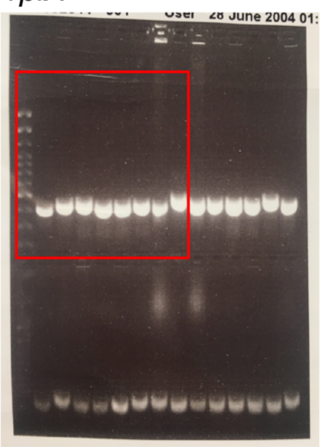

*rplW*

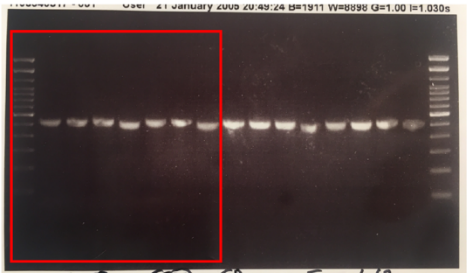

Figure S4d

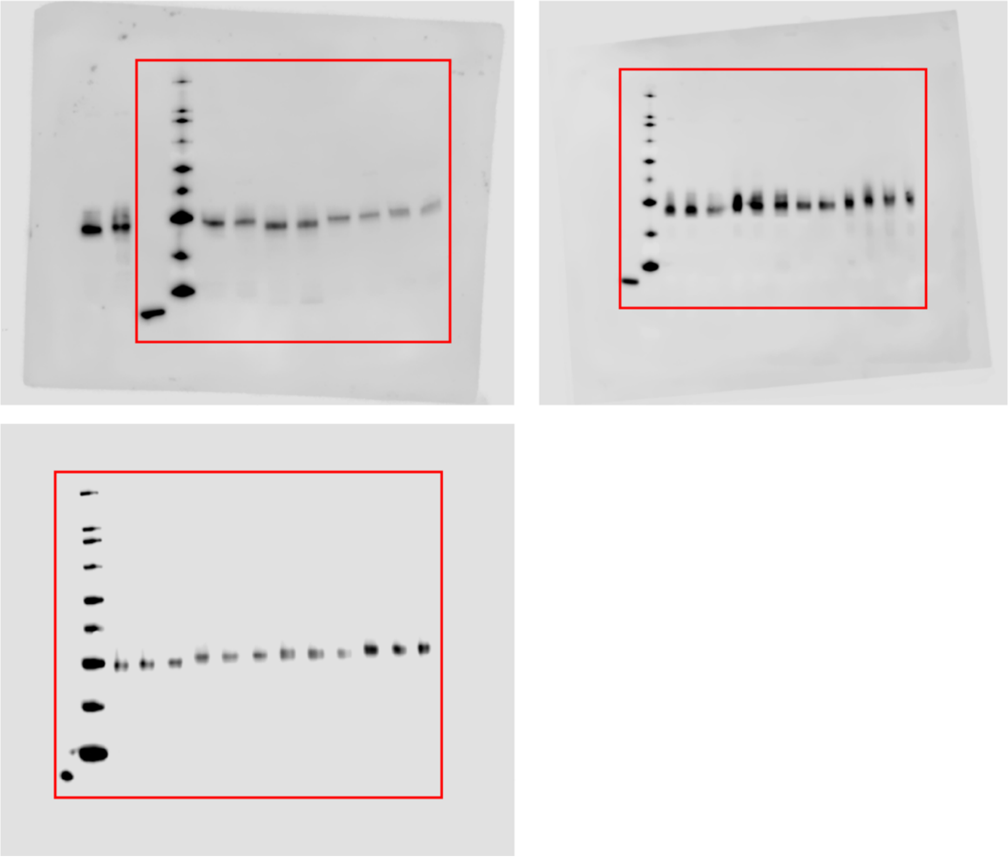

Figure S5c

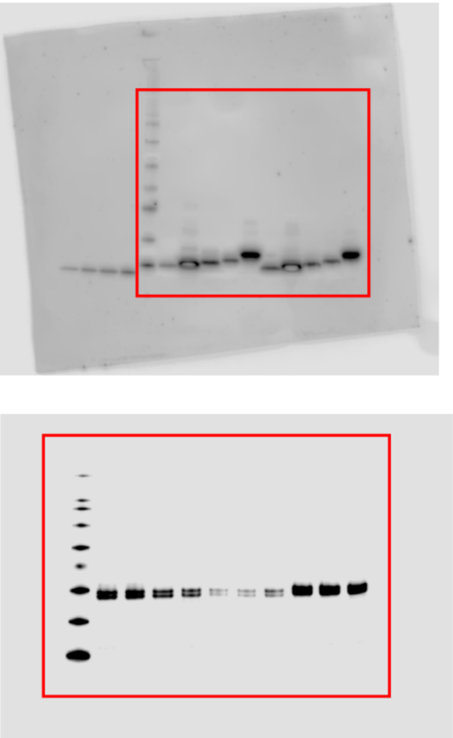

Figure S5e

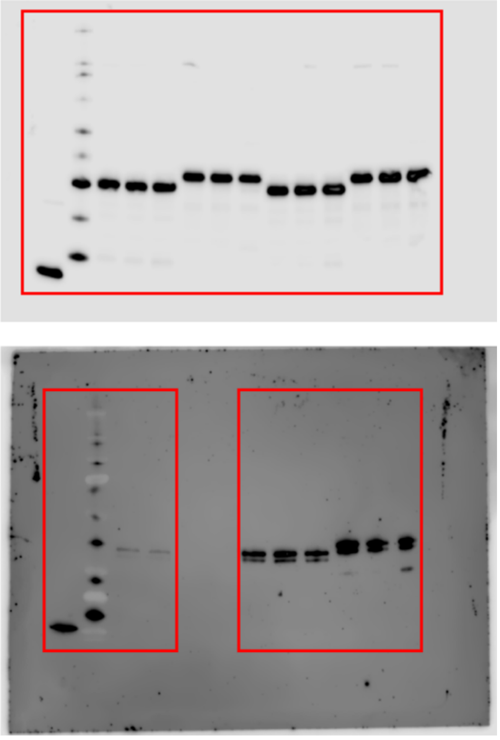

Figure S7b

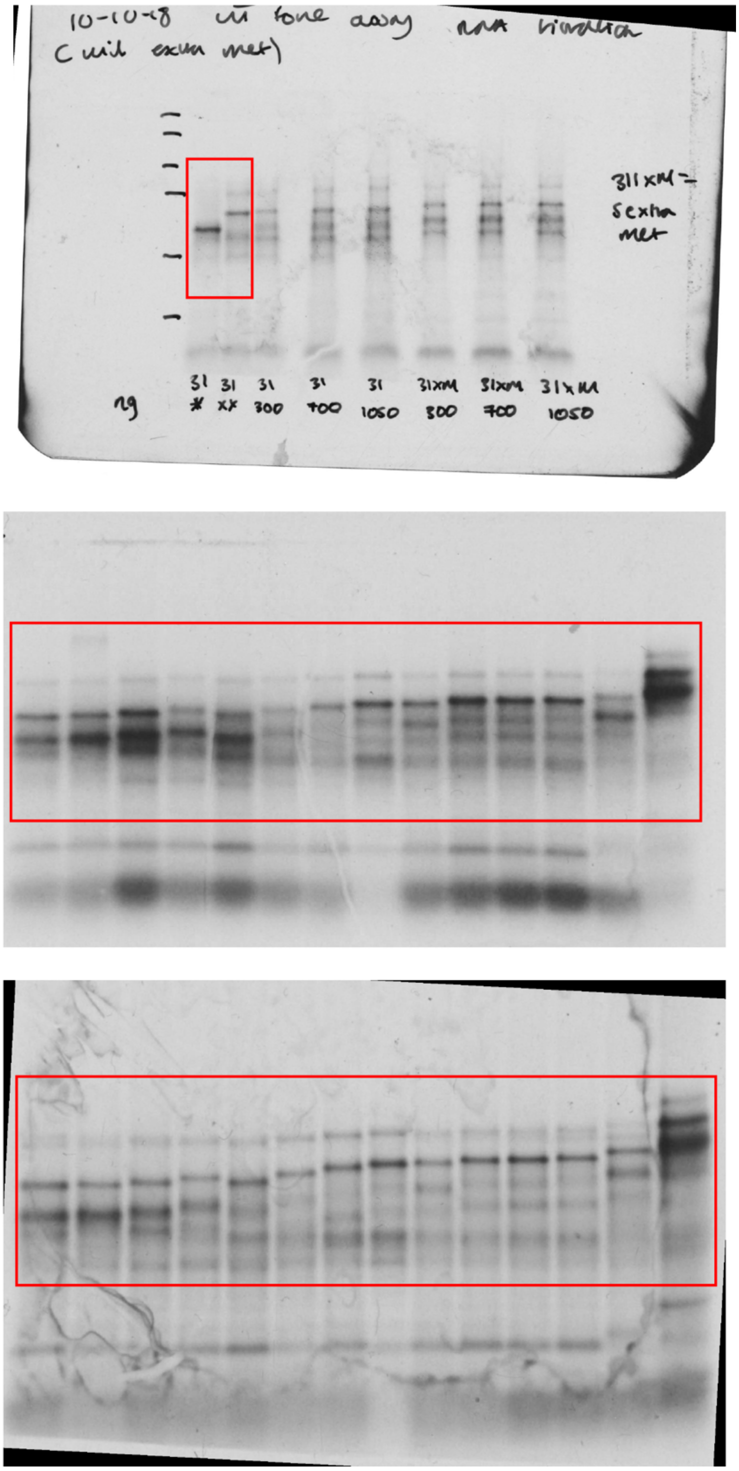

## Supplementary References


1. Yamada KD, Tomii K, Katoh K. Application of the MAFFT sequence alignment program to large data—reexamination of the usefulness of chained guide trees. *Bioinformatics* **32**, 3246-3251 (2016).

2. Webb B, Sali A. Comparative protein structure modeling using MODELLER. *Current protocols in bioinformatics* **54**, 5.6. 1-5.6. 37 (2016).

3. Cabrita LD*, et al.* A structural ensemble of a ribosome–nascent chain complex during cotranslational protein folding. *Nature structural & molecular biology* **23**, 278 (2016).

4. Cassaignau AM*, et al.* Interactions between nascent proteins and the ribosome surface inhibit co-translational folding. *Nature Chemistry*, 1-7 (2021).
