# Supplementary material for: Modulating co-translational protein folding by rational design and ribosome engineering
Source: Nat Commun. 2022 Jul 22;13:4243. doi: 10.1038/s41467-022-31906-z (PMC9307626; doi:10.1038/s41467-022-31906-z)
Supplement: Supplementary file 3 — Description of Additional Supplementary Files [file 41467_2022_31906_MOESM3_ESM.pdf]

File name: Supplementary Movie 1

Description: Movie of NC ensembles of WT RNC (<i>L</i>=37) from all-atom MD simulations. Movie of NC ensembles of WT+37 and $23^{\Delta L}24^{\Delta L}$+37 RNCs from all-atom MD simulations.

File name: Supplementary Movie 2

Description: Movie of NC ensembles of 23^{ΔL}24^{ΔL} RNC (<i>L</i>=37) from all-atom MD simulations. Movie of NC ensembles of WT+37 and $23^{\Delta L}24^{\Delta L}$+37 RNCs from all-atom MD simulations.
