# Supplementary material for: Modulating co-translational protein folding by rational design and ribosome engineering
Source: Nat Commun. 2022 Jul 22;13:4243. doi: 10.1038/s41467-022-31906-z (PMC9307626; doi:10.1038/s41467-022-31906-z)
Supplement: Supplementary file 6 — Reporting Summary [file 41467_2022_31906_MOESM6_ESM.pdf]

nature portfolio

Corresponding author(s): John Christodoulou

Last updated by author(s): Jun 21, 2022

Reporting Summary

Nature Portfolio wishes to improve the reproducibility of the work that we publish. This form provides structure for consistency and transparency in reporting. For further information on Nature Portfolio policies, see our Editorial Policies and the Editorial Policy Checklist.

Statistics

For all statistical analyses, confirm that the following items are present in the figure legend, table legend, main text, or Methods section.

n/a	Confirmed	
☐	☒	The exact sample size (*n*) for each experimental group/condition, given as a discrete number and unit of measurement
☐	☒	A statement on whether measurements were taken from distinct samples or whether the same sample was measured repeatedly
☒	☐	The statistical test(s) used AND whether they are one- or two-sided<br>*Only common tests should be described solely by name; describe more complex techniques in the Methods section.*
☒	☐	A description of all covariates tested
☒	☐	A description of any assumptions or corrections, such as tests of normality and adjustment for multiple comparisons
☐	☒	A full description of the statistical parameters including central tendency (e.g. means) or other basic estimates (e.g. regression coefficient) AND variation (e.g. standard deviation) or associated estimates of uncertainty (e.g. confidence intervals)
☒	☐	For null hypothesis testing, the test statistic (e.g. F, t, r) with confidence intervals, effect sizes, degrees of freedom and P value noted<br>*Give P values as exact values whenever suitable.*
☒	☐	For Bayesian analysis, information on the choice of priors and Markov chain Monte Carlo settings
☒	☐	For hierarchical and complex designs, identification of the appropriate level for tests and full reporting of outcomes
☒	☐	Estimates of effect sizes (e.g. Cohen's *d*, Pearson's *r*), indicating how they were calculated

Our web collection on statistics for biologists contains articles on many of the points above.

Software and code

Policy information about availability of computer code

Data collection	NMR data were recorded using Topspin 3.5pl2, pulse sequences available on https://github.com/chriswaudby/pp. sgRNAs for CRISPR were designed by CRISPOR (4.99). CryoEM data were imported to Relion (3.0/3.1) for processing. MD simulations with structure-based potential were performed using SMOG (2.0) in GROMACS (4.5.7). uL23 and uL24 loop truncations were modeled by Modeler (10.2).
Data analysis	NMR data were analysed using MATLAB (R2019b, The MathWorks Inc.), code available upon request. MultiSeq in VMD (1.9.3) was used for structure-based sequence alignment. Ribosomal protein loop sequences were aligned by MAFFT (7.490). Western blots were analysed by ImageJ (1.53a). CryoEM particles were picked by Gautomatch (v0.56) or crYOLO (1.8.4), and contrast transfer estimation was performed by CTFFIND (4.1). The initial models were rigid-body fitted by UCSF Chimera (1.16). The models were adjusted using COOT (v9) and the modified protein loops were refined using Phenix. The volume of exit tunnel was calculated by POVME (3.0). MD simulations were analysed using Plumed (2.8.0), MDAnalysis (2.2.0) and VMD.

For manuscripts utilizing custom algorithms or software that are central to the research but not yet described in published literature, software must be made available to editors and reviewers. We strongly encourage code deposition in a community repository (e.g. GitHub). See the Nature Portfolio guidelines for submitting code & software for further information.

Data

Policy information about availability of data

All manuscripts must include a data availability statement. This statement should provide the following information, where applicable:

- Accession codes, unique identifiers, or web links for publicly available datasets
- A description of any restrictions on data availability
- For clinical datasets or third party data, please ensure that the statement adheres to our policy

All the data used in the Main and Supplementary figures are supplied in the Source Data file. CryoEM structures are deposited in the PDB and their accession codes and the links are provided.

Field-specific reporting

Please select the one below that is the best fit for your research. If you are not sure, read the appropriate sections before making your selection.

☒ Life sciences ☐ Behavioural & social sciences ☐ Ecological, evolutionary & environmental sciences

For a reference copy of the document with all sections, see nature.com/documents/nr-reporting-summary-flat.pdf

Life sciences study design

All studies must disclose on these points even when the disclosure is negative.

Sample size	For NMR of RNCs, n ≥ 2 for the WT and CRISPR-engineered ribosomes at L=31 for the precise measurements of low populations of the folded state FLN5 (19F) and the CCR rates of the unfolded state FLN5 (15N). Spectra of the samples that show strong intensity of the peaks of interest with high signal-to-noise (S/N) ratio (>>10) were recorded using a single sample (n=1), as typical for solution NMR. All samples undergo rigorous biochemical and NMR quality control measurements, as described and representative data shown in figures. n > 500,000 particles used for cryo-EM data processing after 2D classification. n ≥ 2 for all RNCs for APF assay.
Data exclusions	No data were excluded.
Replication	The repeats of multiple RNC NMR samples (isotopically-labeled either by 15N or 19F) show negligible errors compared to their S/N ratio. The repeats of CRISPR/Cas9 colony PCR show similar success rates with small variations (< 10%). The repeats of AP Force assay show only negligible difference from the reported data (within the error).
Randomization	Randomization was used in CRISPR/Cas9 colony PCR when screening successful transformants, as there is no obvious phenotypic effects of the gene editing on the ribosomal proteins that we generated.
Blinding	Blinding is not applicable in the NMR and other biochemical assays in this manuscript, as the nature of the study was to reveal the structure-function relationship between our rationally designed ribosomes and the behaviour of their nascent chain. Also, this is typical for NMR and structural biology studies.

Reporting for specific materials, systems and methods

We require information from authors about some types of materials, experimental systems and methods used in many studies. Here, indicate whether each material, system or method listed is relevant to your study. If you are not sure if a list item applies to your research, read the appropriate section before selecting a response.

Materials & experimental systems

n/a	Involved in the study	
☐	☒	Antibodies
☒	☐	Eukaryotic cell lines
☒	☐	Palaeontology and archaeology
☒	☐	Animals and other organisms
☒	☐	Human research participants
☒	☐	Clinical data
☒	☐	Dual use research of concern

Methods

n/a	Involved in the study	
☒	☐	ChIP-seq
☒	☐	Flow cytometry
☒	☐	MRI-based neuroimaging

Antibodies

Antibodies used	Anti-6His Mouse-monoclonal Antibody-HRP conjugate (Invitrogen, MA1-21315-HRP. lot WK337821)

Validation	Western blot visualisation as described in the manufacturer's website (https://www.thermofisher.com/order/genome-database/dataSheetPdf?producttype=antibody&productsubtype=antibody_primary&productId=MA1-21315-HRP&version=233).
